# Medical Inspection of Schools

**Published:** 1909-12

**Authors:** 


**Medical Inspection of Schools.** By A. H. HOGARTH, M.B., B.Ch.(Oxon.), D.P.H. Pp. 360. London: Oxford Medical Publications. 1909.

This is a timely and useful handbook, written by the right man. It is not only of value to school medical officers and to all medical practitioners interested in the training and health of school children, but to teachers also, and certain chapters on organisation and administration might well be studied by all school managers and members of Education Committees.

In many countries, notably Sweden, Norway and Argentina, followed by Switzerland, Germany and the United States, schemes dealing more or less effectively with the complex problem of school hygiene have gradually been evolved, generally springing from local municipal effort, later becoming centralised ; but in England there were few centres in which any serious attention had been given to the matter, when the Education (Administrative Provisions) Act of 1907 suddenly imposed upon every local Education Authority the duty of providing for the systematic medical inspection of all children in the elementary schools of England and Wales.

How unprepared the country was may be judged by the fact that " it was not until the Act was actually placed on the Statute Book that a medical officer was appointed to advise the Board of Education, and the first official memorandum was issued only five weeks before local authorities were required to institute a system of medical inspection."

For some years past the Education Committee of the London County Council had been building up a department of school hygiene, and to the medical officers working there, and at Bradford, and a few other of the northern towns, attention was naturally directed for light and leading.

Dr. A. H. Hogarth is one of this band of pioneer workers, and, under the able direction of Dr. Kerr, he has gained an insight into the problem educational authorities as well as the medical profession perforce must face, entitling him to speak on it with authority.

Though attention to school hygiene is now compulsory, it is well, in view of the general lack of knowledge of and preparation for the work to be done, that he has devoted much space in the early part of his book to a review of its history in this and other countries and legislation respecting it, and to a detailed consideration of the " Case for Medical Inspection."

He claims that there is a "national duty to arrange for the classification of children according to their mental capacities, and to adapt the educational system to the requirements of the several groups of children in order to diminish the present economic wastage of misdirected educational efforts." A most desirable goal, but of how difficult attainment can easily be realised by those who know the difficulties and complexities of school management in a large city.

If the school doctor is to give advice as to the adaptation of the educational system to specially selected groups, the local educational authorities, who already have troubles enough, must at least be satisfied that the doctor is not only a medical man, but one with an intimate knowledge of the curriculum he ventures to revise, of the varied conditions of school life, of the facilities afforded by the school premises, and of the possibilities of variation in view of local conditions.

In a special chapter Dr. Hogarth discusses the school doctor, his qualifications and his duties, reviewing the influences he considers necessary to the moulding of his ideal man. He urges *inter alia* the value of the Diploma of Public Health, but thinks that " for such as intend to confine their attention to school work this examination should be considerably modified," and suggests that " possibly examining bodies will see their way to dividing this examination into two separate parts, or to issuing a separate diploma of School Hygiene." In view of the large number of medical men throughout the country who must in future be engaged in this work, this suggestion might well be considered by the Council of our new University.

With the authority of one possessing an intimate knowledge of both branches of the work, he discusses the important matter of the relationship of the school medical officer with the medical officer of health, and concludes that " school hygiene should be regarded as co-ordinate with, and not subordinate to, public health, and that both branches of State medicine could be administered side by side without fear of friction or of overlapping of function on the part of executive officers."

The chapters on " School Hygiene and State Medicine " and on " Local Organisation of School Hygiene " are particularly valuable, the present state of chaos in the central administration of matters pertaining to public health, due to the absence of a central department of State medicine, being well illustrated in the table he gives on page 97. But this is not a complete statement of the confusion, for Bristol possesses a Watch Committee administering the Food and Drugs Act and the Midwives Act, with police surgeons as executive officers.

The difficult problem of medical treatment of children found defective receives full discussion. It is one of the most important and urgent questions the medical profession has now to face. Dr. Hogarth sums up the factors comprising the complex problem tersely as follows :—

1. The ignorance, apathy and neglect of parents.
2. The inadequacy and inaccessibility of existing institutions.
3. The real poverty of the people, and their inability to pay for efficient treatment.

4. The tedious nature of the treatment required for the specified chronic cases.

5. The general standing of the practitioners in poor districts.

Of the last factor he is very outspoken, and says "in certain matters affecting the public health many members of the medical profession appear quite devoid of conscientiousness. They constantly certify children as free from infectious and contagious disease and as fit to attend school when they are obviously suffering from ringworm or scabies, and sometimes even in the case of diphtheria their action is not beyond suspicion. For example, out of 240 certificates of freedom from ringworm no less than 234 were found to be inaccurate on microscopic examination of specimens from the affected cases."

We have not space to enter into the discussion of the solution of the problem, but note that Dr. Hogarth pronounces emphatically that "without the school clinic the whole system of medical inspection becomes practically fruitless."

The book concludes with a chapter on the common diseases affecting school life, more useful to teachers than doctors for whom this presentment of elementary facts can scarcely be necessary; and there follow a useful bibliography, a reprint of extracts from official documents cognate to the subject, and a good index.

